# Weak Correlation between Nucleotide Variation and Recombination Rate across the House Mouse Genome

**DOI:** 10.1093/gbe/evaa045

**Published:** 2020-02-28

**Authors:** Michael E Kartje, Peicheng Jing, Bret A Payseur

**Affiliations:** Laboratory of Genetics, University of Wisconsin – Madison, Madison

**Keywords:** linked selection, recombination rate, *Mus musculus domesticus*

## Abstract

Positive selection and purifying selection reduce levels of variation at linked neutral loci. One consequence of these processes is that the amount of neutral diversity and the meiotic recombination rate are predicted to be positively correlated across the genome—a prediction met in some species but not others. To better document the prevalence of selection at linked sites, we used new and published whole-genome sequences to survey nucleotide variation in population samples of the western European house mouse (*Mus musculus domesticus*) from Germany, France, and Gough Island, a remote volcanic island in the south Atlantic. Correlations between sequence variation and recombination rates estimated independently from dense linkage maps were consistently very weak (*ρ* ≤ 0.06), though they exceeded conventional significance thresholds. This pattern persisted in comparisons between genomic regions with the highest and lowest recombination rates, as well as in models incorporating the density of transcribed sites, the density of CpG dinucleotides, and divergence between mouse and rat as covariates. We conclude that natural selection affects linked neutral variation in a restricted manner in the western European house mouse.

## Introduction

Natural selection can shape genomic patterns of neutral variation. Under certain conditions, both recurrent selection in favor of beneficial mutations and recurrent selection against deleterious mutations are expected to reduce diversity near targets of selection in the genome (Maynard Smith and Haigh 1974; Charlesworth et al. 1993; Hudson and Kaplan 1995). Reductions in diversity should be more severe when selected and neutral mutations are tightly linked, leading to the prediction that nucleotide variation and recombination rate will be positively correlated across the genome (Kaplan et al. 1989; Hudson and Kaplan 1995). Although a wide variety of species show this correlation (Begun and Aquadro 1992; Tenaillon et al. 2002; Cai et al. 2009; Geraldes et al. 2011; Corbett-Detig et al. 2015), the pattern is not universal (Cutter and Payseur 2013). Evaluation of the relationship between nucleotide diversity and recombination rate in additional species is therefore needed to understand the determinants of contrasting genomic patterns across species (Cutter and Payseur 2013).

## Results and Discussion

To test the prediction that background selection and selective sweeps have generated strong positive correlations between genetic diversity and recombination rate, we analyzed nucleotide variation across the genomes of three population samples of the western European house mouse, *Mus musculus domesticus*, from Gough Island, France (Harr et al. 2016), and Germany (Harr et al. 2016). The genomic imprint left by selection at linked sites varies depending on a population’s distance from equilibrium. In order to capture this variation, we chose populations representing near-equilibrium (France, Germany) and nonequilibrium (Gough Island) demographies. We found totals of 14,852,535, 14,987,676, and 17,856,572 high-quality single nucleotide polymorphisms (SNPs) for Gough Island, France, and Germany populations, respectively (see Materials and Methods). Levels of nucleotide variation, averaged over 1-Mb windows, were higher in France (*θ*_π_* *=* *0.00263; *θ*_w_* *=* *0.00192) and Germany (*θ*_π_* *=* *0.00256; *θ*_w_* *=* *0.00219) and lower on Gough Island (*θ*_π_* *=* *0.00219; *θ*_w_* *=* *0.00181).

We compared levels of nucleotide variation across the genome to local recombination rates estimated from the dense genetic map available for house mice (Cox et al. 2009). This genetic map was estimated from over 3,500 meiotic products genotyped at 10,195 markers. For all analyses, recombination rate was measured over 5-Mb windows. Across genomic window sizes and populations, correlations between recombination rate and *θ*_π_ were positive but very low (Spearman’s *ρ *<* *0.06 in all cases), while *P*-values most often fell below typical significance thresholds (*P *<* *0.05 except in one case; table 1). A similar pattern held for recombination rate and *θ*_w_ across all populations and window sizes (*ρ* ≤ 0.061 for all window sizes; *P* < 0.05 except in two cases; table 1).

**Table 1:** Summary Statistics for Nucleotide Diversity (*θ*_π_) and Watterson's Theta (*θ*_w_)

Population	Window Size	Nucleotide Diversity		Rho	*P*-Value	Watterson's Theta		Rho	*P*-Value
		*Avg*	*SD*			*Avg*	*SD*		
*Gough Isl. (n=14)*	2.5-kb	0.0022	0.0029	0.0046	4.2 × 10^–4^	0.0018	0.0021	0.0079	1.8 × 10^–9^
	5-kb	0.0022	0.0027	0.0086	2.6 × 10^–6^	0.0018	0.0019	0.012	5.1 × 10^–11^
	50-kb	0.0022	0.0020	0.019	2.3 × 10^–4^	0.0018	0.0014	0.021	7.0 × 10^–5^
	100-kb	0.0022	0.0018	0.021	0.0027	0.0018	0.0012	0.025	4.2 × 10^–4^
	500-kb	0.0022	0.0014	0.033	0.027	0.0018	0.00094	0.039	0.0084
	1-Mb	0.0022	0.0011	0.054	0.0095	0.0018	0.00079	0.061	0.0033
*Germany (n=8)*	2.5-kb	0.0026	0.0030	0.0045	5.8 × 10^–4^	0.0022	0.0022	0.0037	0.0046
	5-kb	0.0026	0.0028	0.0082	7.0 × 10^–6^	0.0022	0.0021	0.0066	3.0 × 10^–4^
	50-kb	0.0025	0.0021	0.021	7.1 × 10^–5^	0.0022	0.0015	0.017	0.0013
	100-kb	0.0025	0.0019	0.027	1.3 × 10^–4^	0.0022	0.0014	0.023	8.7 × 10^–4^
	500-kb	0.0025	0.0015	0.035	0.017	0.0022	0.0011	0.028	0.060
	1-Mb	0.0026	0.0012	0.054	0.0095	0.0022	0.00091	0.047	0.024
*France (n=4)*	2.5-kb	0.0027	0.0032	0.0015	0.24	0.0019	0.0021	0.0018	0.16
	5-kb	0.0027	0.0030	0.0047	0.010	0.0019	0.0020	0.0052	0.0046
	50-kb	0.0026	0.0022	0.016	0.0018	0.0019	0.0014	0.016	0.0020
	100-kb	0.0026	0.0020	0.022	0.0015	0.0019	0.0013	0.023	0.00095
	500-kb	0.0026	0.0016	0.031	0.034	0.0019	0.0010	0.031	0.036
	1-Mb	0.0026	0.0013	0.045	0.029	0.0019	0.00085	0.047	0.024

Note.—Spearman’s rank correlation results (*ρ* and corresponding *P*-value) are shown for the correlation between recombination rate and either *θ*_π_ or *θ*_w_.

**Fig. 1.**
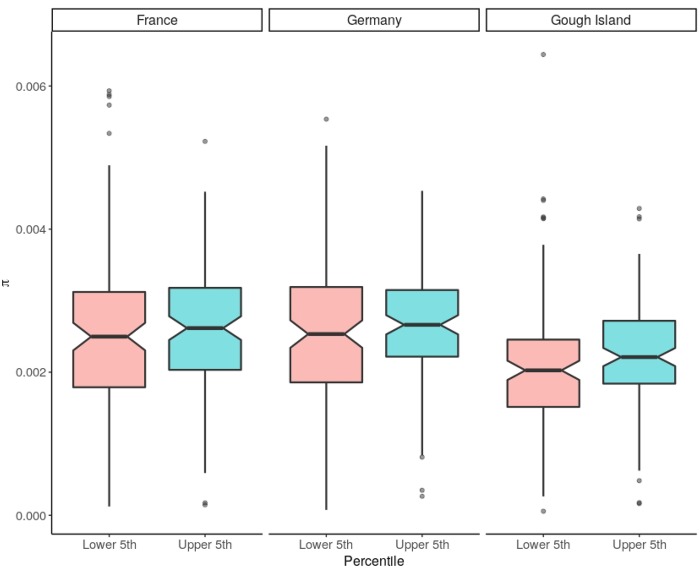
—Nucleotide diversity (*θ*_π_) in windows with recombination rates in either the lower or upper 5th percentiles of the genome-wide distribution. There is no significant difference in *θ*_π_ between high-recombination rate and low-recombination rate windows for France (*P *=* *0.510) or Germany (*P = *0.200). There is a significant difference for Gough Island (*P *=* *0.017) (Wilcoxon rank-sum test).

To mitigate the effects of quantitative uncertainty in recombination rate estimates, we compared nucleotide variation in windows with the highest (95th percentile* *=* *1.20* *cM/Mb) and lowest (5th percentile* *=* *0.14* *cM/Mb) recombination rates in the genome. Consistent with the weak correlations between diversity and recombination rate in the full data set, no significant difference was observed in this contrast for *θ*_π_ in the France or Germany populations (Wilcoxon rank-sum tests; France: *P *=* *0.51; Germany: *P *=* *0.20; fig. 1) or for *θ*_w_ (France: *P *=* *0.58; Germany: *P *=* *0.13) in 1-Mb windows. Gough Island data showed modestly significant *P*-values in this comparison (*θ*_π_: *P = *0.02; *θ*_w_: *P *=* *0.01).

We used two approaches to consider recombination rate variation on a finer genomic scale. First, we took recombination rate estimates from alternative genetic maps generated for chromosomes 1 (Paigen et al. 2008) and 11 (Billings et al. 2010) in house mice (supplementary table 1, Supplementary Material online). On chromosome 1, correlations with nucleotide variation were either nonsignificant or significantly negative at both 5-kb and 1-Mb window sizes, with the exception of *θ*_π_ estimated over 5-kb windows. Chromosome 11 showed weak but significant positive correlations when *θ*_π_ was estimated over 5-kb windows (France: *ρ *=* *0.052, *P *=* *1.08* *×* *10^–^^9^; Germany: *ρ *=* *0.051, *P *=* *1.54* *×* *10^–^^9^; Gough Island: *ρ *=* *0.042, *P *=* *4.18* *×* *10^–^^7^) and no significant correlations were detected when *θ*_π_ was estimated over 1-Mb windows.

We also inspected genomic maps of double-strand break hotspots generated using a variant of chromatin immune-precipitation sequencing (Smagulova et al. 2011; supplementary table 1, Supplementary Material online). Because double-strand breaks are the precursors to crossovers, the landscape of double-strand break hotspots is sometimes used as a surrogate for local recombination rate on the scale of kilobases (Smagulova et al. 2011). We detected no positive correlation between nucleotide diversity and double-strand break hotspot count across 1-Mb windows for France (*ρ *=* *0.0009, *P *=* *0.368), Germany (*ρ *=* *–0.002, *P *=* *0.125), and Gough Island (*ρ *=* *–0.007, *P *=* *1.24* *×* *10^–^^10^). Because 5-kb windows contained at most two double-strand break hotspots, we also compared nucleotide diversity in windows with and without hotspots using Wilcoxon rank-sum tests, under the alternative hypothesis that windows containing hotspots have, on average, higher nucleotide diversity. We found no evidence for a difference (*P *>* *0.19 for all populations).

To search for additional signatures of selection at linked sites while accounting for other factors that could influence nucleotide variation, we analyzed general linear models. We treated nucleotide diversity as the response variable, and recombination rate, the proportion of transcribed sites (a surrogate for the density of selected sites), mouse–rat divergence (a correlate of mutation rate), and the density of CpG dinucleotides (another correlate of mutation rate) as explanatory variables. An interaction term was included in all linear models to account for an observed positive correlation between CpG density and the proportion of transcribed sites across the genome (*ρ *=* *0.541, *P *<* *2.2* *×* *10^–^^16^).

Selection is expected to reduce linked diversity more severely in genomic regions with more selective targets (Barton 1995; Hudson and Kaplan 1995; Payseur and Nachman 2002; Flowers et al. 2012). In linear models that account for covariation with recombination rate, the proportion of transcribed sites negatively influenced *θ*_π_ in both 5-kb windows and 1-Mb windows (table 2). The negative (but weak) relationship between nucleotide diversity and the proportion of transcribed sites was confirmed in bivariate analyses for both 5-kb windows (France: *ρ *=* *–0.065; Germany: *ρ *=* *–0.070; Gough Island: *ρ *=* *–0.075; *P *<* *2.2* *×* *10^–^^16^ for each population) and 1-Mb windows (France: *ρ *=* *–0.052, *P *=* *0.011; Germany: *ρ *=* *–0.050, *P *=* *0.014; Gough Island: *ρ *=* *–0.098, *P *=* *1.70* *×* *10^–^^6^).

**Table 2:** Summary of Linear Models Describing the Effects of Genomic Attributes on Nucleotide Diversity (*θ*_π_)

Window Size	Population	Factor	Estimate	Standard Error	*P*
*1*-Mb	Gough Island (*n* = 14)	Intercept	1.99 × 10^–3^	1.49 × 10^–4^	<2 × 10^–16^
		Recombination rate (cM/Mb)	1.55 × 10^–4^	7.45 × 10^–5^	3.80 × 10^–2^
		Mouse–rat divergence	3.16 × 10^–3^	7.62 × 10^–4^	3.44 × 10^–5^
		CpG density	–8.53 × 10^–8^	3.12 × 10^–8^	6.29 × 10^–3^
		Proportion TX sites	–6.10 × 10^–4^	1.92 × 10^–4^	1.48 × 10^–3^
		CPG density × prop TX sites	–2.94 × 10^–8^	8.98 × 10^–8^	7.43 × 10^–1^
	Germany (*n* = 8)	Intercept	2.20 × 10^–3^	1.65 × 10^–4^	<2 × 10^–16^
		Recombination rate (cM/Mb)	6.95 × 10^–5^	8.21 × 10^–5^	3.97 × 10^–1^
		Mouse–rat divergence	4.09 × 10^–3^	8.40 × 10^–4^	1.23 × 10^–6^
		CpG density	–7.41 × 10^–8^	3.44 × 10^–8^	3.15 × 10^–2^
		Proportion TX sites	–4.79 × 10^–4^	2.11 × 10^–4^	2.35 × 10^–2^
		CPG density × prop TX sites	–3.78 × 10^–8^	9.90 × 10^–8^	7.03 × 10^–1^
	France (*n* = 4)	Intercept	2.47 × 10^–3^	1.71 × 10^–4^	<2 × 10^–16^
		Recombination rate (cM/Mb)	–8.74 × 10^–6^	8.51 × 10^–5^	9.18 × 10^–1^
		Mouse–rat divergence	3.25 × 10^–3^	8.71 × 10^–4^	1.94 × 10^–4^
		CpG density	–8.14 × 10^–8^	3.57 × 10^–8^	2.25 × 10^–2^
		Proportion TX sites	–5.64 × 10^–4^	2.19 × 10^–4^	1.02 × 10^–2^
		CPG density × prop TX sites	2.06 × 10^–8^	1.03 × 10^–7^	8.41 × 10^–1^
*5*-kb	Gough Island (*n* = 14)	Intercept	2.04 × 10^–3^	2.11 × 10^–5^	<2 × 10^–16^
		Recombination rate (cM/Mb)	1.55 × 10^–5^	1.50 × 10^–5^	3.02 × 10^–1^
		Mouse–rat divergence	1.91 × 10^–3^	1.10 × 10^–4^	<2 × 10^–16^
		CpG density	–6.53 × 10^–6^	3.08 × 10^–7^	<2 × 10^–16^
		Proportion TX sites	–5.03 × 10^–4^	5.01 × 10^–5^	<2 × 10^–16^
		CPG density × prop TX sites	–1.12 × 10^–5^	2.11 × 10^–6^	1.07 × 10^–7^
	Germany (*n* = 8)	Intercept	2.37 × 10^–3^	2.24 × 10^–5^	<2 × 10^–16^
		Recombination rate (cM/Mb)	–7.12 × 10^–5^	1.59 × 10^–5^	7.48 × 10^–6^
		Mouse–rat divergence	2.27 × 10^–3^	1.17 × 10^–4^	<2 × 10^–16^
		CpG density	–5.71 × 10^–6^	3.27 × 10^–7^	<2 × 10^–16^
		Proportion TX sites	–3.92 × 10^–4^	5.32 × 10^–5^	1.62 × 10^–13^
		CPG density × prop TX sites	–1.79 × 10^–5^	2.24 × 10^–6^	1.26 × 10^–15^
	France (*n* = 4)	Intercept	2.54 × 10^–3^	2.34 × 10^–5^	<2 × 10^–16^
		Recombination rate (cM/Mb)	–1.26 × 10^–4^	1.66 × 10^–5^	2.97 × 10^–14^
		Mouse–rat divergence	1.85 × 10^–3^	1.22 × 10^–4^	<2 × 10^–16^
		CpG density	–5.12 × 10^–6^	3.42 × 10^–7^	<2 × 10^–16^
		Proportion TX sites	–3.99 × 10^–4^	5.56 × 10^–5^	7.35 × 10^–13^
		CPG density × prop TX sites	–1.74 × 10^–5^	2.34 × 10^–6^	1.29 × 10^–13^

Note.—*θ*_π_ was computed over both 5-kb and 1-Mb window sizes for all populations. TX, Transcribed.

Genomic regions with higher mutation rates are expected to harbor more neutral diversity (Kimura 1983). Consistent with this prediction, mouse–rat divergence was positively correlated with *θ*_π_ in all populations at both 5-kb and 1-Mb window sizes (table 2). CpG density negatively affected *θ*_π_ in all populations for 5-kb windows and 1-Mb windows (table 2).

**Fig. 2.**
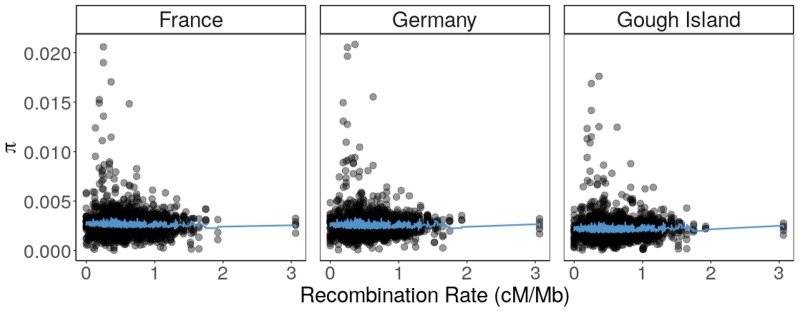
—Nucleotide diversity (*θ*_π_) computed over 1-Mb intervals plotted against recombination rate (cM/Mb) across the autosomal genome. Fitted values (blue line) were obtained from the multiple linear regression of *θ*_π_ against recombination rate and other genomic covariates. The effect size of recombination rate is not statistically significant for France (*P *=* *0.918) or Germany (*P = *0.397), but is significant for Gough Island (*P = *0.038).

Accounting for effects of the proportion of transcribed sites, mouse–rat divergence, and CpG density, higher recombination rate was associated with lower *θ*_π_ in mice from Germany (β_recombination_* *=* *–7.12* *×* *10^–^^5^; *P *=* *7.48* *×* *10^–^^6^) and France (β_recombination_* *=* *–1.26* *×* *10^–^^4^; *P *=* *2.97* *×* *10^–^^14^) for 5-kb windows (fig. 2), but not in mice from Gough Island (*P *=* *0.302; fig. 2). In 1-Mb windows, recombination rate did not affect *θ*_π_ in France (*P *=* *0.918) or Germany (*P *=* *0.397), but was modestly significant for Gough Island (β_recombination_* *=* *1.55* *×* *10^–^^4^, *P *=* *0.038) (fig. 2).

Our demonstration that recombination rate and nucleotide variation are only weakly correlated extends similar findings in house mice (Geraldes et al. 2011) to the genome-wide level. What explains this weak relationship? It seems unlikely that sequencing error is responsible. Sequencing depth was moderate to high (>10×, on average; Harr et al. 2016) and levels of nucleotide variation were similar to those observed using Sanger sequencing of a smaller number of loci in other population samples of *M. m. domesticus* (Geraldes et al. 2011). We expect recombination rate estimates to be accurate because the Cox et al. (2009) genetic map surveyed a large number of meioses (3,546). Recombination rates can evolve (Smukowski and Noor 2011; Dapper and Payseur 2017a), so estimates from contemporary genetic maps might differ from recombination rates during the history of the samples we studied. Nevertheless, the *rank order* of recombination rates across the genome should be mostly conserved on this timescale. The lack of differences in sequence variation between genomic regions with very high- and very low-recombination rate and the similarity of results using other maps argues against uncertainty in or evolution of recombination rates as causes of our results. Booker et al. (2017) uncovered a similarly weak correlation between nucleotide diversity and recombination rate in another subspecies of house mice (*M. m. castaneus*) using fine-scale recombination rate estimates based on linkage disequilibrium.

The weak relationship between nucleotide variation and recombination rate across the genome suggests that the effects of selection on linked neutral diversity are modest in house mice. Several biological factors can reduce the strength and pervasiveness of selection at linked sites (Cutter and Payseur 2013). The absence of a correlation between nucleotide variation and recombination rate in rice was attributed to a relatively higher density of selective targets in regions of high-recombination (Flowers et al. 2012). The proportion of transcribed sites was positively correlated with recombination rate in our analysis (*ρ *=* *0.178; *P *<* *2.2* *×* *10^–^^16^), suggesting that the genomic arrangement of mutations that affect fitness could similarly dampen the signature of selection at linked sites in house mice. Both models in which linked diversity is reduced by recurrent positive selection (“genetic hitchhiking”; Maynard Smith and Haigh 1974; Stephan et al. 1992; Wiehe and Stephan 1993) and models in which diversity is reduced by recurrent purifying selection (“background selection”; Charlesworth et al. 1993) usually assume demographic equilibrium. Population bottlenecks in the three populations of house mice we surveyed (Gray et al. 2014; Harr et al. 2016) could mask selection at linked sites (Beissinger et al. 2016), though such histories are expected to amplify selective signatures in some cases (Torres et al. 2018). In contrast to positive selection on new mutations, selection targeting standing variation and/or spread across a large number of variants is not predicted to strongly reduce linked diversity (Hermisson and Pennings 2005; Pritchard et al. 2010; Stephan 2019); perhaps, the genetic architecture of adaptation is mostly polygenic and dominated by standing variants in house mice. The distribution of selection coefficients in house mice might not fall within the parameter space that generates pervasive selection signatures in levels of neutral diversity. Recent theoretical studies have shown that purifying selection against recessive mutations may affect linked variation in a similar manner to associative overdominance, potentially masking signatures of background selection (Zhao and Charlesworth 2016; Becher et al. 2020; Gilbert et al. 2020). Finally, it is possible that background selection and genetic hitchhiking affect linked variation, but the density of selective targets is too small and the effects too localized (Booker and Keightley 2018) to generate strong correlations between nucleotide variation and recombination rate across the genome. Regardless of the explanation, our findings serve as a reminder that the effects of selection at linked sites can vary in important ways among species.

## Materials and Methods

Population genomic analyses were conducted using three geographically distant populations of house mice. Wild mice (*n *=* *14) were collected from Gough Island during September 2009 (Gray et al. 2014). High molecular weight DNA was extracted from liver tissue using Qiagen DNeasy blood and tissue DNA extraction kits (Gray et al. 2014). DNA concentration and sizing were verified using the Qubit dsDNA HS Assay Kit (Life Technologies, Carlsbad, CA, USA) and Agilent DNA 1000 chip (Agilent Technologies, Inc., Santa Clara, CA, USA), respectively. Samples were prepared according to the TruSeq PCR Free Sample Preparation kit (Illumina Inc., San Diego, CA, USA) with minor modifications. Libraries were selected for an average insert size of 550 bp using SPRI-based bead selection. Quality of the finished libraries was assessed using the Kapa Illumina NGS Library Quantification Kit (KAPA Biosystems, Wilmington, MA, USA). Libraries were standardized to 2 nM. Cluster generation was performed using the Illumina Rapid PE Cluster Kits v2 and the Illumina cBot. Paired-end, 100 bp sequencing was performed, using Rapid v2 SBS chemistry on an Illumina HiSeq2500 sequencer at the University of Wisconsin-Madison Biotechnology Center. Images were analyzed using the Illumina Pipeline, version 1.8.2. Libraries were sequenced to an average of 11.66× coverage per sample. Quality control was performed on raw read data using FASTQC (https://www.bioinformatics.babraham.ac.uk/projects/fastqc/, last accessed March 13, 2020). Filtered, trimmed reads were then aligned to the mm10 house mouse genome assembly using BWA-MEM v.0.7.10 (Li and Durbin 2009). Raw read data from Harr et al. (2016) for France (*n *=* *4) and Germany (*n *=* *8) populations were aligned using the same procedure. Variant calling was performed for SNPs from the pooled set of alignments for each population using GATK HaplotypeCaller v3.7-0-gcfedb67 (McKenna et al. 2010) to produce a variant call format (VCF) file containing SNP calls from all populations. Only variants with a Phred-scaled quality score ≥100 were included in subsequent analyses.

Levels of sequence variation within populations were estimated using two common summary statistics, nucleotide diversity (*θ*_π_) (Nei and Li 1979; Tajima 1983) and the number of segregating sites adjusted for expected coalescence time (*θ*_w_) (Watterson 1975). *θ*_π_ is the average number of pairwise differences between sequences, and $\theta_{w}=k/a$ (where *k* is the number of segregating sites at a locus, and *a* is the expected coalescence time of a sample in units of the effective population size, $\sum_{i=1}^{n-1}1/i$). For each population, per-bp *θ*_π_ and per-bp *θ*_w_ were computed from a filtered VCF over 2.5-kb, 5-kb, 50-kb, 100-kb, 500-kb, and 1-Mb windows using a custom Python script. To focus on putatively neutral polymorphisms, only nontranscribed sites were included in calculations of *θ*_π_ and *θ*_w_. Python and R code used to conduct analyses are available at https://github.com/mekartje/mmdom_SALS.

To date, several studies examining the association between recombination rate and diversity have estimated recombination rates from patterns of linkage disequilibrium. While this approach offers the advantage of high-genomic resolution, it can be misled by assuming demographic equilibrium (Li and Stephens 2003; Dapper and Payseur 2017b) and the absence of selective sweeps (Reed and Tishkoff 2006). To obtain estimates of recombination rates independent of diversity summaries, recombination rates were computed from three published genetic maps. The recombination rates primarily used in this study were estimated from a genome-wide linkage map generated from a heterogeneous stock of *M. m. domesticus* (Cox et al. 2009). Patterns of recombination rate variation were verified using rates estimated from independent linkage maps of *M. m. domesticus* chromosomes 1 (Paigen et al. 2008) and 11 (Billings et al. 2010). Each of the three maps was constructed from crosses involving large numbers of house mice, featuring many meioses. We estimated recombination rate as the slope of the linear regression of genetic map position (cM) against physical position (Mb) for all markers included in each 5-Mb interval of the genetic map. We estimated the density of selective targets by calculating the proportion of transcribed sites in a window. To assign transcription status, genome feature data were obtained from the UCSC genome browser annotations for the mm10 mouse genome assembly.

To account for effects of mutation rate on nucleotide diversity, we computed divergence between mouse and rat, and the density of CpG dinucleotides. Mouse–rat divergence was estimated from a chained and netted whole-genome alignment between mm10 and rn6 genome assemblies downloaded from the UCSC genome browser (https://genome.ucsc.edu/cgi-bin/hgGateway, last accessed March 13, 2020). We used the Jukes–Cantor correction to account for multiple hits (Jukes and Cantor 1969). CpG density was computed by counting the frequency of CpG dinucleotides across the mm10 genome.

At all window sizes, the association between recombination rate and nucleotide variation was first examined using the nonparametric Spearman’s rank correlation. To evaluate the effect of recombination rate on nucleotide diversity in the context of other genetic covariates, we fit linear models with nucleotide diversity as the response variable, and recombination rate, the proportion of transcribed sites, mouse–rat divergence, and CpG dinucleotide density as explanatory variables. Linear models were fit to diversity estimates obtained from 5-kb and 1-Mb window sizes. Because of a significant rank correlation between CpG density and the proportion of transcribed sites, we included a term for the interaction between these two variables in all linear models. By comparing effect sizes and significance values of model covariates, the explanatory power of recombination rate was evaluated relative to other sources of variation in nucleotide diversity.

## Supplementary Material

evaa045_Supplementary_Data

## Notes

**Data deposition:** All raw read data for Gough Island are available via the NCBI Sequence Read Archive (BioProject accession PRJNA587779).
